# Rebooting effective clinical supervision practices to support healthcare workers through and following the COVID-19 pandemic

**DOI:** 10.1093/intqhc/mzac030

**Published:** 2022-04-15

**Authors:** Priya Martin, Saravana Kumar, Esther Tian, Geoff Argus, Srinivas Kondalsamy-Chennakesavan, Lucylynn Lizarondo, Tiana Gurney, David Snowdon

**Affiliations:** Rural Clinical School, Faculty of Medicine, The University of Queensland, Toowoomba, QLD 4350, Australia; Darling Downs Health, Baillie Henderson Hospital, Toowoomba, QLD 4350, Australia; Allied Health and Human Performance, University of South Australia, Adelaide, SA 5000, Australia; Allied Health and Human Performance, University of South Australia, Adelaide, SA 5000, Australia; Southern Queensland Rural Health, Faculty of Health and Behavioural Sciences, The University of Queensland, 152 West St, South Toowoomba, QLD 4350, Australia; Rural Clinical School, Faculty of Medicine, The University of Queensland, Toowoomba, QLD 4350, Australia; Rural Clinical School, Faculty of Medicine, The University of Adelaide, Adelaide, SA 5005, Australia; Rural Clinical School, Faculty of Medicine, The University of Queensland, Toowoomba, QLD 4350, Australia; JBI, Monash University, Melbourne, VIC 3199, Australia

**Keywords:** clinical supervision, COVID-19, effectiveness, health personnel, mental health, pandemics

## Abstract

The importance of clinical supervision, a professional support and clinical governance mechanism, to patients, healthcare workers and organizations has been well documented. Clinical supervision has been shown to support healthcare workers during challenging times, by reducing burnout, enhancing mental health and wellbeing at work, and improving job satisfaction. However, clinical supervision participation and effectiveness are pre-requisites for realising these benefits. During times of stress and increased workloads (e.g. during the Coronavirus pandemic), healthcare workers tend to prioritise clinical duties and responsibilities over clinical supervision. Effective supervision practices can be restored, and healthcare workers can be better supported in their roles during and in the post-pandemic period only if healthcare workers, policy makers, healthcare organizations, clinical supervision trainers and researchers join forces. This paper sheds light on this important topic and offers a number of practical recommendations to reboot effective clinical supervision practices at the point of care.

## Background

Healthcare workers globally have borne the brunt of the impact caused by the Coronavirus (i.e. COVID-19) pandemic. The higher risks of psychological distress and fatigue-facing healthcare workers is expected to adversely impact them, their organizations and communities [1]. Two scoping reviews [2, 3] on the physical and mental health impacts of COVID-19 on healthcare workers, have reported high levels of depression, anxiety, insomnia and distress. Female healthcare workers, nurses and those engaging in direct COVID-19 patient care were found to be disproportionally affected and at a higher risk of experiencing these symptoms [2], although men may under-report these issues. Accordingly, there are calls for the implementation of well-being measures to tackle impending healthcare worker burnout and psychological morbidities to combat the COVID-19 pandemic and its impacts [1–3].

Pre-COVID-19 research already showed that doctors were at a higher risk of mental health issues. A pre-pandemic large-scale Australian survey of over 14 000 doctors and medical students found substantially higher rates of psychological distress and suicidal thoughts in this group compared to the broader population and other professionals [4]. The COVID-19 pandemic is likely to exacerbate the work-related stress and mental health issues that they already face.

The importance of support measures for healthcare workers (including nurses, doctors and allied health professionals), during these unprecedented times, is being echoed around the globe [1]. While new measures are being called for to better support healthcare workers, it is imperative to also identify and leverage existing mechanisms such as clinical supervision where healthcare workers and students receive support in their roles from more experienced colleagues [5]. Effective clinical supervision can support healthcare workers through times of stress and organizational change and have beneficial effects through the reduction of burnout and enhanced work satisfaction [6]. Clinical supervision has three functions: formative (developing skills and knowledge), normative (administrative, day-to-day matters) and restorative (supportive) [7], with the restorative functions likely to be of most help in the post-pandemic period as it can enhance healthcare worker mental health and well-being [5].

**Table 1:** ENGAGE Strategies

Call for action	Strategies
Examine	We call upon healthcare and professional organizations to urgently examine current clinical supervision practices of their employees and develop and implement measures to ensure participation and adherence to evidence-informed supervision practices.
Normalize	We call upon policymakers to strengthen existing clinical supervision policies and guidelines that would facilitate and normalize healthcare workers prioritizing their clinical supervision, including telesupervision or distance supervision.
Gather	We call upon healthcare workers to gather and attend to their mental health and well-being by prioritizing clinical supervision. We recommend using a clinical supervision model such as the Proctor’s model that incorporates supportive functions within clinical supervision, which is crucial in the current context. We urge healthcare workers, especially those considered to be at a higher risk of experiencing mental health issues (i.e. doctors, nurses and female healthcare workers) to make use of support avenues provided by employers including clinical supervision. This is even more important for recent graduates, those new in their roles, and those that have had a change in duties/responsibilities owing to the pandemic.
Access	We encourage healthcare workers, especially those in new or changed roles, to seek out clinical supervision and ensure their supervisor is the best fit to meet their new/revised learning goals.
Grow	We call upon training providers to grow evidence-informed training opportunities on enhancing the effectiveness of clinical supervision, evidence-informed telesupervision and restorative clinical supervision.
Evaluate	We call upon clinical supervision researchers to evaluate current clinical supervision practices, any changes implemented and resulting outcomes to ensure that these strategies deliver the outcomes they set out to achieve. We encourage researchers to undertake targeted research on telesupervision of healthcare workers as this area continues to remain outdated and under-researched.

For healthcare workers to benefit from clinical supervision, there are two pre-cursors: participation in and effectiveness of clinical supervision [8, 9]. A recent systematic review of the impact of clinical supervision on healthcare organizational outcomes found that healthcare organizations can reap the benefits of clinical supervision (e.g. reduced burnout, improved staff satisfaction at work), only when the clinical supervision provided and the clinical supervisor are effective [9]. When confronted with clinical priorities and a burgeoning clinical load, healthcare workers (i.e. both supervisors and supervisees) are likely to prioritize the client’s welfare over their own, forgoing strategies aimed at their own well-being (such as clinical supervision) [5]. In other words, just when they need the most support, they are likely to receive none. Recent studies on the impact of the pandemic on clinical supervision of healthcare workers and students on placements have confirmed this revealing that when supervisees sought more support, they did not receive it. Studies have further highlighted that the pandemic has adversely impacted several parameters of clinical supervision (e.g. frequency, duration). Findings also indicate that those supervisees who had supervisors that were well-supported in their roles, displayed consistent supervisory behaviours, were more satisfied with the clinical supervision they received. This reinforces that healthcare workers that are well-supported are able to provide better support to other junior staff. Recent studies have further confirmed the mental health and well-being concerns experienced by healthcare workers in their roles [10–12].

## Recommendations

It is in this context that clinical supervision, a readily existing accessible tool, can support healthcare workers, if set up and delivered effectively. We provide some recommendations to strengthen and restore effective clinical supervision practices at the point of care. We expect the strategies outlined to be applicable across a wide variety of professions and healthcare contexts (Table 1).

Healthcare organizations, healthcare workers, managers, researchers and training providers can all play a role in implementing the ENGAGE recommendations provided. Organizations can embed these strategies in policies and processes which will ensure a system-wide approach which are not dependent on local drivers and individuals. Healthcare workers, both supervisors and supervisees, can make ENGAGE a part of routine conversations around clinical supervision so it is embedded as part of local vocabulary and culture. Training providers can incorporate restorative clinical supervision and telesupervision components in training to facilitate healthcare worker skill development in these areas. A systems-level approach that is embedded as part of routine practice and complemented with ongoing training is more likely to be impactful and sustainable.

It is now widely acknowledged that the effects of this pandemic will continue to have an impact for years to come. In order to ameliorate these long-lasting effects, it is crucial to strengthen supportive mechanisms such as clinical supervision to better care for those who care for others. As the saying goes, ‘we cannot pour from an empty cup’, we call upon healthcare workers to safeguard their own mental health and wellbeing during these unprecedented times, to be able to care for others.
